# Association between subjective physical function and occurrence of new fractures in older adults: A retrospective cohort study

**DOI:** 10.1111/ggi.14830

**Published:** 2024-02-17

**Authors:** So Sato, Yusuke Sasabuchi, Shotaro Aso, Akira Okada, Hideo Yasunaga

**Affiliations:** ^1^ Department of Clinical Epidemiology and Health Economics, Graduate School of Medicine The University of Tokyo Tokyo Japan; ^2^ Department of Real‐world Evidence, Graduate School of Medicine The University of Tokyo Tokyo Japan; ^3^ Department of Prevention of Diabetes and Lifestyle‐Related Diseases, Graduate School of Medicine The University of Tokyo Tokyo Japan

**Keywords:** health check‐ups, late‐stage elderly questionnaire, new fractures, predictive abilities, subjective physical function

## Abstract

**Background:**

The Late‐Stage Elderly Questionnaire has been incorporated into health assessments for older adults in Japan, encompassing three self‐administered questions on subjective physical function: subjective gait speed decline, recent fall history, and exercise habits. Nevertheless, its efficacy in predicting new fracture occurrences remains uncertain.

**Methods:**

This retrospective cohort study utilized Japan's DeSC database, a large commercially available claims database. Participants were older adults aged ≥75 years and provided complete responses to the Late‐Stage Elderly Questionnaire at health check‐ups. We performed two Cox regression analyses for new fractures based on the responses to the three questions (Model 1) and on age, sex, and responses to the three questions (Model 2). The predictive abilities of the 1‐year occurrence of new fractures were compared between the two models.

**Results:**

Of 11 683 eligible older adults, 927 (7.93%) experienced new fractures. Model 1 revealed significant associations between new fractures and subjective gait speed decline (hazard ratio [HR], 1.63; 95% confidence interval [CI], 1.40–1.89), recent fall history (HR, 2.03; 95% CI, 1.77–2.33), and absence of exercise habits (HR, 1.29; 95% CI, 1.13–1.47). Model 2 demonstrated superior predictive ability (area under the curve, 0.677; 95% CI, 0.659–0.695) compared with Model 1 (area under the curve, 0.633; 95% CI, 0.614–0.652), with a net reclassification improvement of 0.383 (95% CI, 0.317–0.449).

**Conclusion:**

Three subjective physical well‐being factors were significantly associated with new fracture development in older adults. These results suggest that the three‐question assessment may be a valuable screening tool for identifying new fractures. **Geriatr Gerontol Int 2024; 24: 337–343**.

## Graphical abstract

This study evaluated the Japanese Late‐Stage Elderly Questionnaire's efficacy in predicting new fractures among those aged ≥75 years. New fractures were linked to gait speed decline, recent fall history, and a lack of exercise habits. The model combining age, sex, and questionnaire responses showed superior predictive ability, implying its potential as a screening tool.

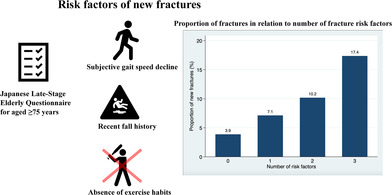

## Introduction

Population aging is a global trend,^1^ and Japan exhibits the second highest aging rate (29.1%) among nations experiencing rapid demographic aging.^2^ To improve the health of older adults, Japan introduced the Late‐Stage Elderly Questionnaire into health checkups for adults aged ≥75 starting in 2020.^3^ This questionnaire was developed to assess comprehensively the health status of older adults, taking into account factors such as frailty.^4^ Within the questionnaire's Physical Function and Falls section, three inquiries pertain to subjective physical function: a decline in subjective gait speed, a history of falls within the past year, and exercise habits. The objective of this section is to identify individuals at risk of fractures and falls, which are the fourth leading cause of long‐term care needs in Japan.^5^

Previous studies have reported that older adults are less inclined to possess exercise routines,^6^ that their gait speed diminishes with advancing age,^7^ and that a reduction in gait speed is associated with an elevated likelihood of requiring long‐term care within 2 years.^8^ In addition, older adults often exhibit factors that heighten their susceptibility to falls, such as cognitive impairment, the use of sleep‐inducing medications, and polypharmacy.^9^ The guidelines of the Centers for Disease Control and Prevention (CDC) and those of the American/British Geriatrics Society guidelines advocate screening for fall risk using 12 questions,^10^, ^11^ and a previous study evaluated the predictive efficacy of the CDC‐recommended 12 questions.^12^

However, the Late‐Stage Elderly Questionnaire assesses subjective physical function using only three questions. Nevertheless, it remains uncertain whether these three variables independently correlate with the occurrence of new fractures. Furthermore, it remains unclear whether this Japanese three‐question evaluation is effective in predicting the occurrence of new fractures, whereas other countries commonly employ the CDC‐endorsed 12‐question subjective assessment.

Consequently, our objective was to clarify the association between the three factors related to subjective physical function within the Late‐Stage Elderly Questionnaire and the occurrence of fractures during the ensuing year. In addition, we examined the capacity of the three‐question assessment to anticipate the occurrence of new fractures.

## Methods

### Data source

This study used the DeSC database (DeSC Healthcare, Inc.), a repository comprising commercially available administrative claims and health‐checkup data. Comprehensive details regarding this database are provided elsewhere.^13^, ^14^

The database includes health insurance claims data from three types of health insurers: (i) national health insurance for non‐employees and individual proprietors; (ii) health insurance for large corporate employees; and (iii) the Advanced Elderly Medical Service System for those aged ≥75. Consequently, the DeSC database includes individuals from a range of age groups, including young, middle‐aged, and older adults. The DeSC database contains information on approximately 12 000 000 individuals, and its age distribution closely mirrors that of Japanese population estimates.^14^ Medical claims data for both outpatients and inpatients are anonymized and maintained at the individual level. These claims data include the following information: (i) a unique identifier; (ii) birth month and sex; (iii) diagnoses coded according to the International Classification of Diseases, 10th Revision (ICD‐10) codes; (iv) procedures based on the original Japanese identification system; (v) pharmaceutical dispensations recorded using the Anatomical Therapeutic Chemical (ATC) Classification System; and (vi) dates of insurance enrollment and disenrollment.

Furthermore, the database includes health checkup data. During their annual health checkups, adults aged ≥75 years completed the Late‐Stage Elderly Questionnaire. This self‐administered questionnaire was organized into 10 categories: (i) health status, (ii) mental health status, (iii) dietary habits, (iv) oral function, (v) weight change, (vi) physical function and falls, (vii) cognitive function, (viii) smoking, (ix) social engagement, and (x) social support.^4^ The physical function and falls section of the questionnaire included the following three questions on subjective physical function: “Do you perceive a decline in gait speed compared to before?”; “Have you experienced any falls in the past year?”; and “Do you engage in exercise, such as walking, at least once a week?”.^5^

### Study design and participant selection

This retrospective cohort study used the data collected between April 2014 and August 2021. The index date for each individual was defined as the day of their initial health checkup during the observation period. The inclusion criteria consisted of individuals who (i) underwent health check‐ups for adults aged ≥75 years, (ii) provided complete responses to the physical function and falls section of the Late‐Stage Elderly Questionnaire, and (iii) were enrolled in the DeSC database within 1 year before the health check‐up, thereby enabling a retrospective 1 year observation period preceding the index date. We excluded individuals who: (i) were diagnosed with malignant neoplasms of bone and articular cartilage (ICD‐10 codes: C40–C41, D480), multiple myeloma and malignant plasma cell neoplasms (C90), or secondary malignant neoplasm of bone and bone marrow (C795); (ii) sustained any pathological fractures (M484, M80, M844), stress fractures (M484, M843), or bone fractures related to neoplastic diseases (M907); (iii) underwent cardiopulmonary resuscitation owing to risk of rib fractures^15^; or (iv) presented incomplete body mass index data. Eligible individuals were tracked from the index date to the occurrence of new fractures, disenrollment in the DeSC database, death, termination, or conclusion of the study, or 1 year following the index date, whichever transpired first.

### Exposure of interest

A positive response to the question, “Do you perceive a decline in gait speed compared to before?” was defined as a subjective gait speed decline. An affirmative response to the question “Have you experienced any falls in the past year?” was defined as a history of falls within the past year. Conversely, a negative response to the question “Do you engage in exercise, such as walking, at least once a week?” was defined as a lack of exercise.

### Outcomes and covariates

The primary outcome in this study was the identification of new bone fractures within 1 year of the health checkup, as determined by ICD‐10 codes (Table S1).^15^

Covariates included age (75–79, 80–84, 85–89, 90–94, ≥95 years), sex, body mass index (<18.5, 18.5–25.0, 25.0–30.0, ≥30.0 kg/m^2^), smoking status (current or former smoker), Charlson comorbidity index (0–2, ≥3),^16^ the number of prescribed medications reflecting polypharmacy (0–4: non‐polypharmacy, 5–9: polypharmacy, ≥10: hyper‐polypharmacy),^17^ comorbidities related to falls or bone fractures, medications related to falls or bone fractures, and any rehabilitation. The variables included in the analyses were based on previous studies of falls and fractures.^15^, ^18^, ^19^, ^20^, ^21^, ^22^, ^23^, ^24^, ^25^, ^26^, ^27^ Table S2 provides additional details on these variables. In line with a previous study,^17^ we extracted data for 88 types of frequently prescribed medications, or those with a potential for causing adverse drug events in older persons. Of these, we excluded short‐acting non‐benzodiazepine hypnotics and nitric acid medicines because these are not covered by Japanese public health insurance, resulting in a total of 86 categories (Table S3). We determined polypharmacy by calculating the number of prescribed drug classes for each individual from the 86 available categories. Comorbidities related to falls or bone fractures were identified if they occurred within 365 days before the index date, while the number of prescribed medications was determined within 30 days before the index date.^28^

### Statistical analysis

The baseline characteristics were summarized and compared across the occurrence of new bone fractures using the chi‐squared test.

For the primary analysis, we conducted a Cox regression analysis to assess the correlation between responses to the three questions and new fractures within 1 year (Model 1). Given that only age and sex were known before the health check‐up, another Cox regression analysis was conducted, including age and sex as covariates (Model 2).

In a secondary analysis, we examined the predictive capabilities of Models 1 and 2 for new fractures within 1 year using Cox regression analyses and receiver operating characteristic curves. The models were compared by evaluating the area under the curve. To evaluate the degree of improvement in the predictive ability for new fractures, the net reclassification improvement was employed to quantify the effect of the added predictors.^29^

Next, we calculated the proportion of new fractures based on the number of positive responses to the three questions.

For the sensitivity analyses, we defined a new fracture as one that occurs in a different location from a previous fracture. Then, we repeated the same analyses with the modified fracture definition.

The threshold for statistical significance was set at *P* = 0.05. All statistical analyses were performed using Stata SE software (version 17.0; StataCorp).

## Results

**Figure 1:**
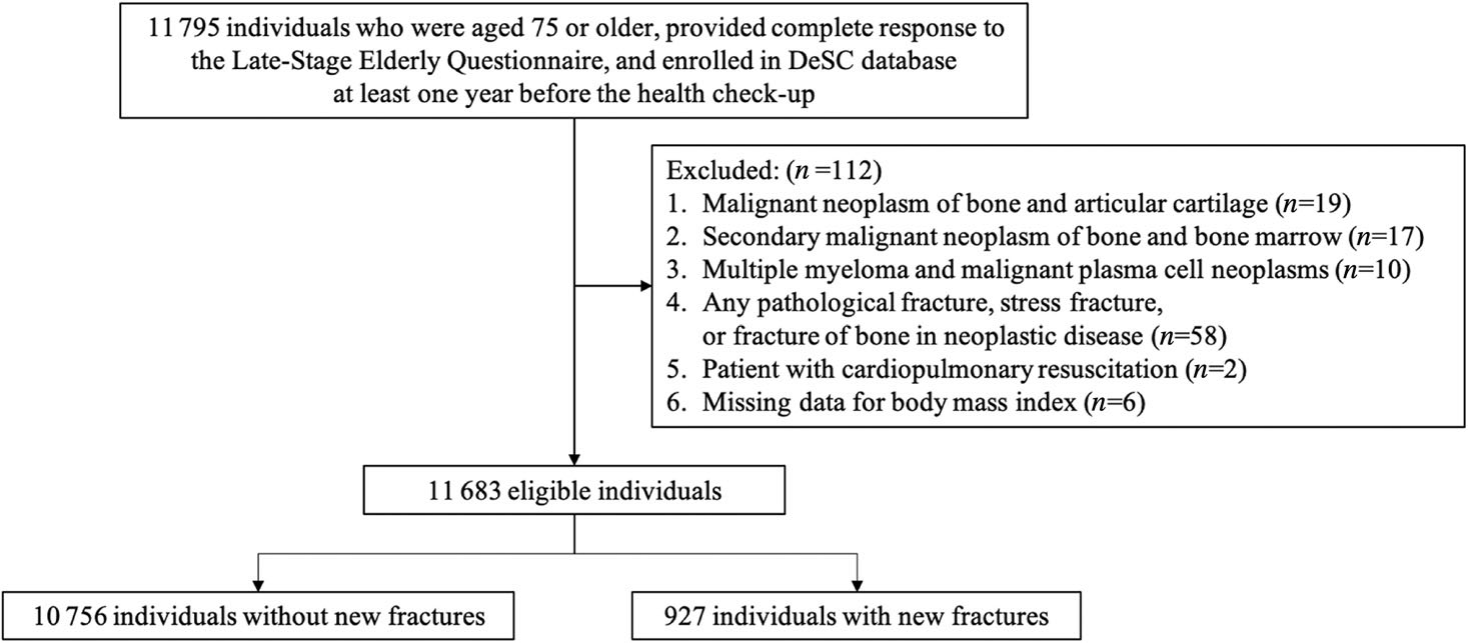
Flowchart of the selection of study participants.

Following the application of the inclusion and exclusion criteria, 11 683 older adults were identified between April 2014 and August 2021 (Fig. 1). New fractures occurred in 927 (7.93%) individuals, and 42 (0.36%) died. The median follow‐up duration was 91 days (interquartile range 55–312; mean 168; standard deviation 142). Of 3455 person‐years of follow‐up, the incidence rate of new fractures was 263 per 1000 person‐years (95% confidence interval (CI), 246 to 281). The detailed information on bone fractures identified by ICD‐10 codes in the participants with new fractures is shown in Table S4.

Table 1 displays the baseline characteristics of older adults with and without new fractures. Older adults who experienced new fractures exhibited a significantly higher percentage of individuals with a decline in subjective gait speed, a history of falls within the past year, and no exercise habits. Furthermore, those who experienced new fractures were older and had a higher Charlson Comorbidity Index score, polypharmacy, comorbidities (excluding chronic obstructive pulmonary disease and chronic kidney disease), medication use (excluding antidiabetic drugs), and rehabilitation utilization compared with those without fractures. In contrast, older adults who developed new fractures had a significantly lower proportion of males and a lower proportion of past or current smokers compared with those without fractures.

**Table 1:** Patient characteristics

	Without new fractures *N* = 10 756	With new fractures *N* = 927	*P*‐value
	*n* (%)	*n* (%)	
Age, years			
75–79	5597 (52.0)	399 (43.0)	<0.001
80–84	3434 (31.9)	312 (33.7)	
85–89	1367 (12.7)	170 (18.3)	
90–94	313 (2.9)	41 (4.4)	
≥95	45 (0.4)	5 (0.5)	
Male	5082 (47.2)	255 (27.5)	<0.001
Body mass index, kg/m^2^			
<18.5	732 (6.8)	71 (7.7)	0.038
18.5–24.9	7462 (69.4)	605 (65.3)	
25.0–29.9	2295 (21.3)	231 (24.9)	
≥30.0	267 (2.5)	20 (2.2)	
Charlson comorbidity index			
0–2	8487 (78.9)	683 (73.7)	<0.001
≥3	2269 (21.1)	244 (26.3)	
Smoking habit			
Current smoker	411 (3.8)	28 (3.0)	<0.001
Past smoker	2099 (19.5)	120 (12.9)	
Polypharmacy			
No polypharmacy (0–4)	8798 (81.8)	669 (72.2)	<0.001
Polypharmacy (5–9)	1871 (17.4)	244 (26.3)	
Hyper‐polypharmacy (≥10)	87 (0.8)	14 (1.5)	
Comorbid medical conditions			
Dementia	505 (4.7)	70 (7.6)	<0.001
Alzheimer's disease	420 (3.9)	50 (5.4)	0.027
Delirium	16 (0.1)	6 (0.6)	<0.001
Parkinsonism	195 (1.8)	28 (3.0)	0.010
Hypertension	6876 (63.9)	647 (69.8)	<0.001
Cerebral infarction	1465 (13.6)	176 (19.0)	<0.001
Sequelae of cerebral infarction	892 (8.3)	110 (11.9)	<0.001
Chronic obstructive pulmonary disease	196 (1.8)	20 (2.2)	0.470
Arthritis	719 (6.7)	102 (11.0)	<0.001
Arthrosis	4182 (38.9)	534 (57.6)	<0.001
Rheumatoid arthritis	233 (2.2)	35 (3.8)	0.002
Sarcopenia	74 (0.7)	14 (1.5)	0.005
Osteoporosis	2495 (23.2)	550 (59.3)	<0.001
Epilepsy	174 (1.6)	28 (3.0)	0.002
Chronic kidney disease	451 (4.2)	33 (3.6)	0.350
Drugs			
Antipsychotics	159 (1.5)	20 (2.2)	0.110
Antidepressants	258 (2.4)	49 (5.3)	<0.001
Benzodiazepines	1657 (15.4)	185 (20.0)	<0.001
Other sedatives	189 (1.8)	25 (2.7)	0.041
Vasodilators	337 (3.1)	50 (5.4)	<0.001
Beta‐blockers	687 (6.4)	73 (7.9)	0.078
Diuretics	489 (4.5)	64 (6.9)	0.001
Antidiabetics	855 (7.9)	84 (9.1)	0.230
Glucocorticoids	559 (5.2)	87 (9.4)	<0.001
Osteoporosis drugs	773 (7.2)	224 (24.2)	<0.001
Rehabilitation	1108 (10.3)	228 (24.6)	<0.001
Physical function and falls section of the Late‐Stage Elderly Questionnaire			
Decline in subjective gait speed	6156 (57.2)	674 (72.7)	<0.001
History of falls within the past year	2163 (20.1)	354 (38.2)	<0.001
Absence of exercise habits	3921 (36.5)	439 (47.4)	<0.001

The primary analyses are presented in Table 2. Model 1 demonstrated that new fractures were significantly associated with a decline in subjective gait speed (hazard ratio [HR], 1.63; 95% CI, 1.40–1.89), history of falls within the past year (HR, 2.03; 95% CI, 1.77–2.33), and the absence of exercise habits (HR, 1.29; 95% CI, 1.13–1.47).

**Table 2:** Results of Cox regression analyses of new fractures in Models 1 and 2

	Model 1			Model 2		
	HR	95% CI	*P*‐value	HR	95% CI	*P*‐value
Physical function and falls section of the Late‐Stage Elderly Questionnaire						
Decline in subjective gait speed	1.63	1.40–1.89	<0.001	1.50	1.29–1.74	<0.001
History of falls within the past year	2.03	1.77–2.33	<0.001	1.96	1.71–2.24	<0.001
Absence of exercise habits	1.29	1.13–1.47	<0.001	1.25	1.09–1.42	0.001
Age	NA			1.04	1.03–1.06	<0.001
Male	NA			0.48	0.42–0.56	<0.001

Model 1 (Cox regression analyses for fractures based on the responses to the three questions on physical function and falls section of the Late‐Stage Elderly Questionnaire). Model 2 (Cox regression analyses for fractures based on age, sex, and responses to the three questions on physical function and falls of the Late‐Stage Elderly Questionnaire). CI, confidence interval; HR, hazard ratio; NA, not applicable.

**Figure 2:**
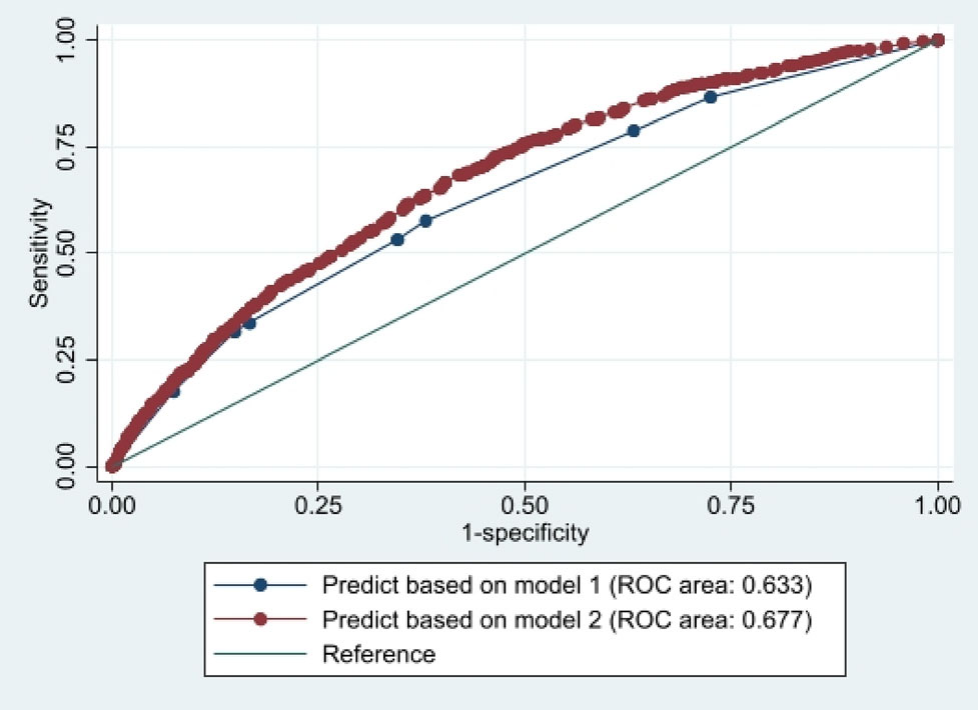
ROC curve of the two models for older adults with a history of fractures. The ROC curve for Model 1 (prediction of fractures based on responses to three questions on the physical function and falls section of the Late‐Stage Elderly Questionnaire) is depicted in blue, and the ROC curve for Model 2 (prediction of fractures based on age, sex, and responses to three questions) is depicted in red. The area under the curve of Models 1 and 2 were 0.63 3 (0.614–0.651) and 0.677 (0.659–0.694), respectively. ROC, receiver operating characteristic.

Figure 2 shows the receiver operating characteristic curves depicting the predictive capacity for new fractures within 1 year based on Models 1 and 2. The area under the curve of Model 2 (0.677; 95% CI, 0.659–0.694) was significantly superior to that of Model 1 (0.633; 95% CI, 0.614–0.652; *P* < 0.001). The incorporation of age and sex significantly improved the predictive ability for new fractures (net reclassification improvement, 0.383; 95% CI, 0.317–0.449; *P* < 0.001).

**Figure 3:**
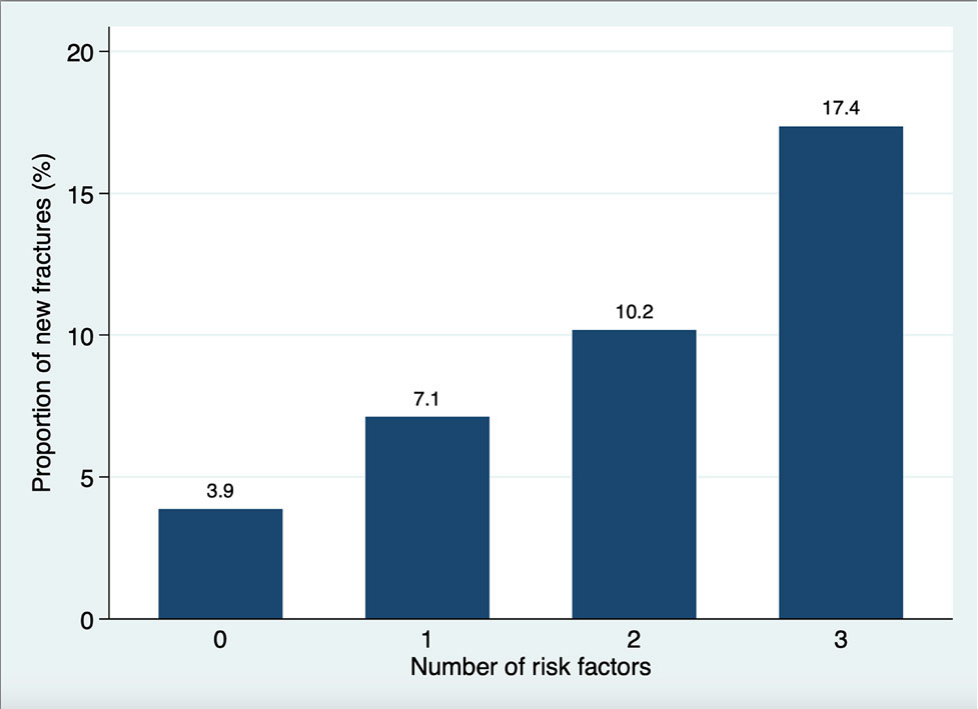
Proportion of fractures in relation to number of fracture risk factors. The absolute risk associated with multiple factors is determined based on the response to three subjective physical function questions in older adults. These risk factors include a decline in subjective gait speed, a history of falls within the past year, and an absence of exercise habits.

Figure 3 depicts the proportion of older adults experiencing new fractures within 1 year based on the number of positive responses to the three questions. The percentage of new fractures increased from 3.9% to 17.4% as the number of positive responses increased.

A new fracture at a different location from a previous fracture was observed in 272 (2.3%) participants. The results of the sensitivity analyses were consistent with those of the primary and secondary analyses (Table S5 and Fig. S1).

## Discussion

This extensive retrospective cohort study demonstrates a significant association between responses to the three questions regarding physical function and falls within the Late‐Stage Elderly Questionnaire and the incidence of new bone fractures within 1 year. Moreover, our study revealed the robust predictive capacity of these questions for new fractures.

The incidence rate of new fractures in this study (263 per 1000 person‐years) appears slightly higher than that reported in previous studies on falls and fractures.^30^, ^31^, ^32^ These previous studies indicated that one‐third of older adults aged ≥65 years experienced falls annually,^30^ with 20% of these falls resulting in fall‐related injuries.^31^, ^32^ In addition, the incidence of falls and fractures increased with age.^32^ The slightly higher incidence rate of new fractures in this study could be attributed to the older age of participants compared with previous studies, where the lowest age was 75 years instead of 65 years.

Previous studies established that having a history of falls within the past year and a lack of exercise habits were significantly associated with the occurrence of new fractures.^31^, ^33^ However, no previous studies have shown that a decline in subjective gait speed is significantly connected with the incidence of new fractures. To the best of our knowledge, this is the first study to establish that a decline in subjective gait speed is significantly associated with new fractures. Moreover, our secondary analysis underscores the strong predictive capability of responses to the three questions. The predictive ability of Model 2 was found to be superior to that of Model 1. Therefore, when screening for new fractures, it is recommended to incorporate age and sex into the three questions. Our findings suggest that a model incorporating age, sex, and responses to the three questions can predict fractures within 1 year. Further research is warranted to evaluate interventions aimed at preventing fractures.

The CDC's screening, with its 12 questions and various objective checks to assess the risk of falls, may be burdensome for older adults. Two simplified versions, each comprising three key questions and several objective assessments, have been developed, with a previous study revealing their predictive ability for identifying future falls within 6 months.^12^ Conversely, the simplified three‐question format of the Japanese Late‐Stage Elderly Questionnaire may offer broader potential as a screening tool, considering the greater response burden associated with the CDC's 12 questions. The prominent difference between Japanese and CDC screenings is the approach used to evaluate the decline in gait speed, with the Japanese assessment relying on subjective evaluation and the CDC screening employing objective measures. This study shows the significant association between a decline in subjective gait speed and new fractures. Given the simplicity of subjectively assessing gait speed decline in comparison with objective evaluation, it may have broader potential for utilization in the screening of new fractures. Balancing the number of screening procedures with prediction accuracy presents a trade‐off, warranting future investigations to compare the screening burden and the predictive accuracy between the Japanese Late‐Stage Elderly Questionnaire and the CDC‐recommended assessment.

The primary objectives of the Late‐Stage Elderly Questionnaire and the CDC's tool are different, with the former screening for fractures and the latter focusing on new falls. This distinction may be crucial because the consequences of fractures can be more severe than the falls themselves for older adults. Therefore, the Japanese tool, which assesses the risk of fractures, may potentially be more beneficial. For example, a previous review indicated that hip fractures could lead to mortality in 14%–36% of cases, and up to 20% might lose their ability to walk altogether. As a result, about 10%–60% might not be able to return to their homes.^34^ These findings highlighted the life‐changing impact of fractures on older adults.

Our study has several limitations. First, health checkups are not mandatory, and healthier individuals may be more inclined to undergo them, potentially resulting in selection bias. Second, our study exclusively included Japanese residents; therefore, the generalizability of the results to other nations may be constrained. Third, the DeSC database lacks information on the occurrence of new falls. Therefore, it may not have been possible to distinguish between past fractures and new fractures. However, considering that the results of the sensitivity analyses, which investigated the occurrence of new fractures in locations different from past fractures, were similar to our primary findings, we believe that our results are robust.

In conclusion, our study underscores the significant association between the three questions concerning physical function and falls within the Late‐Stage Elderly Questionnaire and the occurrence of new fractures. The inclusion of age, sex, and responses to these questions in a predictive model proves effective in anticipating new fractures. Our findings advocate for the simplicity and broader utilization of fall and fracture screening during health checkups for older adults in Japan.

## Author contributions

SS designed the research. SS, YS, and AO conducted the research. SS, YS, and AO analyzed the data. SS, YS, SA, AO, and HY wrote the manuscript. SS was primarily responsible for the final content. All the authors have read and approved the final version of the manuscript.

## Disclosure statement

The authors declare no conflict of interest.

## Ethics statement

This study was approved by the Institutional Review Board of the Graduate School of Medicine at the University of Tokyo. The requirement for written consent was waived owing to data anonymity.

## Supporting information


**Table S1.** Definition of fractures.
**Table S2.** Details of the study covariates.
**Table S3.** Definition of oral medication classes for calculating the number of prescribed drug classes.
**Table S4.** Detailed information on bone fractures identified by ICD‐10 codes in the participants.
**Table S5.** Results of Cox regression analyses of new fractures in Models 1 and 2 (sensitivity analyses).
**Figure S1.** ROC curve of the two models for older adults with a history of fractures (sensitivity analyses). The ROC curve for Model 1 (prediction of fractures based on the responses to three questions on the physical function and falls section of the Late‐Stage Elderly Questionnaire) is depicted in blue, and the ROC curve for Model 2 (prediction of fractures based on age, sex, and the responses to three questions) is depicted in red. The AUC of Models 1 and 2 were 0.594 (0.560–0.628) and 0.627 (0.593–0.661), respectively. The NRI was 0.314; 95% CI 0.194–0.434; *P* < 0.001. AUC, area under the curves; NRI, net reclassification improvement; ROC, receiver operating characteristic.

## Data Availability

The data that support the findings of this study are available from DeSC Healthcare, Inc., Tokyo, Japan. Restrictions apply to the availability of these data, which were used under license for this study. Data are available [https://desc-hc.co.jp/contact] with the permission of DeSC Healthcare, Inc., Tokyo, Japan.
