# Supplementary material for: Association between subjective physical function and occurrence of new fractures in older adults: A retrospective cohort study
Source: Geriatr Gerontol Int. 2024 Feb 17;24(4):337–43. doi: 10.1111/ggi.14830 (PMC11503554; doi:10.1111/ggi.14830)
Supplement: Supplementary file 1 — Table S1. Definition of fractures. Table S2. Details of the study covariates. Table S3. Definition of oral medication classes for calculating the number of prescribed drug classes. Table S4. Detailed information on bone fractures identified by ICD‐10 codes in the participants. Table S5. Results of Cox regression analyses of new fractures in Models 1 and 2 (sensitivity analyses). Figure S1. ROC curve of the two models for older adults with a history of fractures (sensitivity analyses). The ROC curve for Model 1 (prediction of fractures based on the responses to three questions on the physical function and falls section of the Late‐Stage Elderly Questionnaire) is depicted in blue, and the ROC curve for Model 2 (prediction of fractures based on age, sex, and the responses to three questions) is depicted in red. The AUC of Models 1 and 2 were 0.594 (0.560–0.628) and 0.627 (0.593–0.661), respectively. The NRI was 0.314; 95% CI 0.194–0.434; P < 0.001. AUC, area under the curves; NRI, net reclassification improvement; ROC, receiver operating characteristic. [file GGI-24-337-s001.docx]

**Association Between Subjective Physical Function and Occurrence of New Fractures in Older Adults: A Retrospective Cohort Study**

**Supplementary Table S1.** Definition of Fractures.

**Supplementary Table S2.** Details of the Study Covariates.

**Supplementary Table S3.** Definition of Oral Medication Classes for Calculating the Number of Prescribed Drug Classes.

**Supplementary Table S4.** Detailed information on bone fractures identified by ICD-10 codes in the participants.

**Supplementary Table S5.** Results of Cox regression analyses of new fractures in Models 1 and 2 (sensitivity analyses).

**Supplementary Figure S1.** ROC curve of the two models for older adults with a history of fractures (sensitivity analyses).

**Supplemental Table S1.** Definition of Fractures.

| (ICD-10 codes) |
| --- |
| Fracture of skull and facial bones (S02)  Fracture of neck (S12)  Fracture of rib(s), sternum, and thoracic spine (S22)  Fracture of lumbar spine and pelvis (S32)  Fracture of shoulder and upper arm (S42)  Fracture of forearm (S52)  Fracture at wrist and hand level (S62)  Fracture of femur (S72)  Fracture of lower leg including ankle (S82)  Fracture of foot excluding ankle (S92)  Fractures involving multiple body regions (T02)  Fracture of spine (T08)  Fracture of upper limb (T10)  Fracture of lower limb (T12)  Fracture of unspecified body region (T142) |

Abbreviations: ICD, International Classification of Diseases and Related Health Problems, 10th revision

**Supplemental Table S2.** Details of the Study Covariates.

| Patient characteristics | Comorbidities medical conditions related to falls or bone fracture (ICD-10 codes) | Drugs related to falls or bone fracture (ATC classification) | Rehabilitation (Japanese procedure code) |
| --- | --- | --- | --- |
| Age  Sex  Body mass index  Charlson comorbidity index  Smoking habits  Polypharmacy  The response to the questions of physical function and falls section of “Late-Stage Elderly Questionnaire” | Dementia (F00–F03)  Alzheimer’s disease (G30)  Delirium (F05)  Parkinsonism (G20–G26)  Hypertension (I10, I15)  Cerebral infarction (I63)  Sequelae of cerebral infarction (I69)  Chronic Obstructive Pulmonary Disease (J44)  Arthritis (M00, M01, M13)  Arthrosis (M15–M19)  Rheumatoid arthritis (M05, M06, M790)  Sarcopenia (M625)  Osteoporosis (M81, M82)  Epilepsy (G40, G41)  Chronic kidney disease (N18) | Antipsychotics (N05A)  Antidepressants (N06A)  Benzodiazepines (N05BA, N05CD, N05CF)  Other sedatives (N05CA, N05CB, N05CC, N05CE, N05CH, N05CM, N05CX)  Vasodilators (C04A)  Beta-blockers (C07)  Diuretics (C03)  Antidiabetics (A10)  Glucocorticoids (H02AB, H02BX)  Osteoporosis drugs (A12A, G03XC, H05, M05) | Rehabilitation for cerebrovascular disease (H001)  Rehabilitation for motor function disorder (H002)  Rehabilitation for pulmonary disease (H003)  Rehabilitation for cardiovascular disease (H000)  Rehabilitation for disuse syndrome (H001-2)  Rehabilitation for neoplasm (H007-2) |

Abbreviations: ICD, International Classification of Diseases and Related Health Problems, 10th revision code; ATC, Anatomical therapeutic chemical.

**Supplemental Table S3**. Definition of Oral Medication Classes for Calculating the Number of Prescribed Drug Classes.

| Category | Class of medication |
| --- | --- |
| Antipsychotic drugs and hypnotics | Benzodiazepine anxiolytic |
|  | Ultrashort-acting benzodiazepine hypnotic |
|  | Short-acting benzodiazepine hypnotic |
|  | Middle- to long-acting benzodiazepine hypnotic |
|  | Ultrashort-acting non-benzodiazepine hypnotic |
|  | Barbiturate |
|  | Diazepam |
|  | Melatonin receptor agonist |
|  | Orexin receptor antagonist |
|  | Benzamide |
|  | Butyrophenone |
|  | Phenothiazine |
|  | Serotonin dopamine antagonist |
|  | Multi-acting receptor-targeted antipsychotic |
|  | Dopamine system stabiliser |
|  | Dopamine partial agonist |
|  | Lithium carbonate |
|  | 5-HT1A agonist |
|  | Tricyclic antidepressant |
|  | Tetracyclic antidepressant |
|  | Selective serotonin reuptake inhibitor |
|  | Serotonin noradrenaline reuptake inhibitor |
|  | Noradrenergic and specific serotonergic antidepressant |
|  | Others |
| Antidementia drugs | Donepezil |
|  | Galantamine |
|  | Memantine |
| Anticoagulants and antiplatelets | Antiplatelet |
|  | Anticoagulant |
|  | Direct oral anticoagulant |
| Hypotensive drugs and diuretics | Ca2+ blocker (excluding verapamil) |
|  | Angiotensin II receptor blocker |
|  | Angiotensin-converting enzyme inhibitor |
|  | α-blocker |
|  | β-blocker |
|  | Sympatholytic |
|  | Loop diuretic |
|  | Spironolactone |
|  | Thiazide |
|  | Others, including combination drugs |
| Antiarrhythmic drugs | Class 1a |
|  | Class 1b |
|  | Class 1c |
|  | Class 3 |
|  | Class 4 |
|  | Digoxin |
|  | Verapamil |
| Hyperlipemia medicines | Statin |
|  | Fibrate |
|  | Ezetimibe |
|  | Eicosapentaenoic acid |
|  | Nicotinic acid |
|  | Others, including combination drugs |
| Gastrointestinal drugs | Proton pump inhibitor |
|  | Potassium-competitive acid blocker |
|  | H2 antagonist |
|  | Muscarinic antagonist |
|  | Prostaglandin analogue |
|  | Sucralfate |
|  | Others, including combination drugs |
| Diabetes therapeutic drugs | Biguanide |
|  | Thiazolidine |
|  | Dipeptidyl peptide-4 inhibitor |
|  | Sulfonylurea |
|  | Glinide |
|  | α-glucosidase inhibitor |
|  | Sodium-glucose cotransporter 2 inhibitor |
|  | Others, including combination drugs |
| Analgesics | Acetaminophen |
|  | Non-steroidal anti-inflammatory drug |
|  | Cyclooxygenase inhibitor |
|  | Pregabalin |
|  | Tramadol |
|  | Others, including combination drugs |
| Japanese herbal medicines | All herbal medicines prescribed |
| Enteral nutrition | All enteral nutrition (drugs only) |
| Other drugs | Anticholinergic drug |
|  | L-Dopa |
|  | α-blocker |
|  | 5α-reductase inhibitor |
|  | Oxybutynin |
|  | Muscarinic antagonist |
|  | α-blocker |
|  | Antiemetic |
|  | Prochlorperazine maleate |
|  | H1 antagonist |

**Supplementary Table S4.** Detailed information on bone fractures identified by ICD-10 codes in the participants.

| ICD-10 codes | With new fractures  N=927^*^ |
| --- | --- |
| Fracture of skull and facial bones (S02) | 11 |
| Fracture of neck (S12) | 13 |
| Fracture of rib(s), sternum, and thoracic spine (S22) | 265 |
| Fracture of lumbar spine and pelvis (S32) | 286 |
| Fracture of shoulder and upper arm (S42) | 54 |
| Fracture of forearm (S52) | 91 |
| Fracture at wrist and hand level (S62) | 30 |
| Fracture of femur (S72) | 86 |
| Fracture of lower leg including ankle (S82) | 55 |
| Fracture of foot excluding ankle (S92) | 31 |
| Fractures involving multiple body regions (T02) | 21 |
| Fracture of spine (T08) | 20 |
| Fracture of upper limb (T10) | 0 |
| Fracture of lower limb (T12) | 0 |
| Fracture of unspecified body region (T142) | 197 |

Abbreviations: ICD, International Classification of Diseases and Related Health Problems, 10th revision code

* Because older adults could be diagnosed with multiple ICD-10 codes, the total ICD-10 code’s diagnosis exceeds the number of older adults with fractures.

**Supplementary Table S5.** Results of Cox Regression Analyses of New Fractures in Models 1 and 2 (sensitivity analyses).

|  | Model 1 | |  | Model 2 | |  |
| --- | --- | --- | --- | --- | --- | --- |
|  | HR | 95% confidence interval | p-value | HR | 95% confidence interval | p-value |
| Physical function and falls section of the Late-Stage Elderly Questionnaire. |  |  |  |  |  |  |
| Decline in subjective gait speed. | 1.58 | 1.21–2.06 | 0.001 | 1.48 | 1.13–1.93 | 0.004 |
| History of falls within the past year. | 1.52 | 1.17–1.97 | 0.002 | 1.47 | 1.13–1.92 | 0.004 |
| Absence of exercise habits. | 1.33 | 1.04–1.69 | 0.022 | 1.28 | 1.00–1.63 | 0.050 |
| Age | NA |  |  | 1.04 | 1.01–1.06 | 0.010 |
| Male | NA |  |  | 0.57 | 0.44–0.74 | <0.001 |

Abbreviations: HR, hazard ratio; NA, not applicable.

Model 1 (Cox regression analyses for fractures based on the responses to the three questions on physical function and falls section of the Late-Stage Elderly Questionnaire).

Model 2 (Cox regression analyses for fractures based on age, sex, and responses to the three questions on physical function and falls of the Late-Stage Elderly Questionnaire).

**Supplementary Figure S1.** ROC curve of the two models for older adults with a history of fractures (sensitivity analyses).


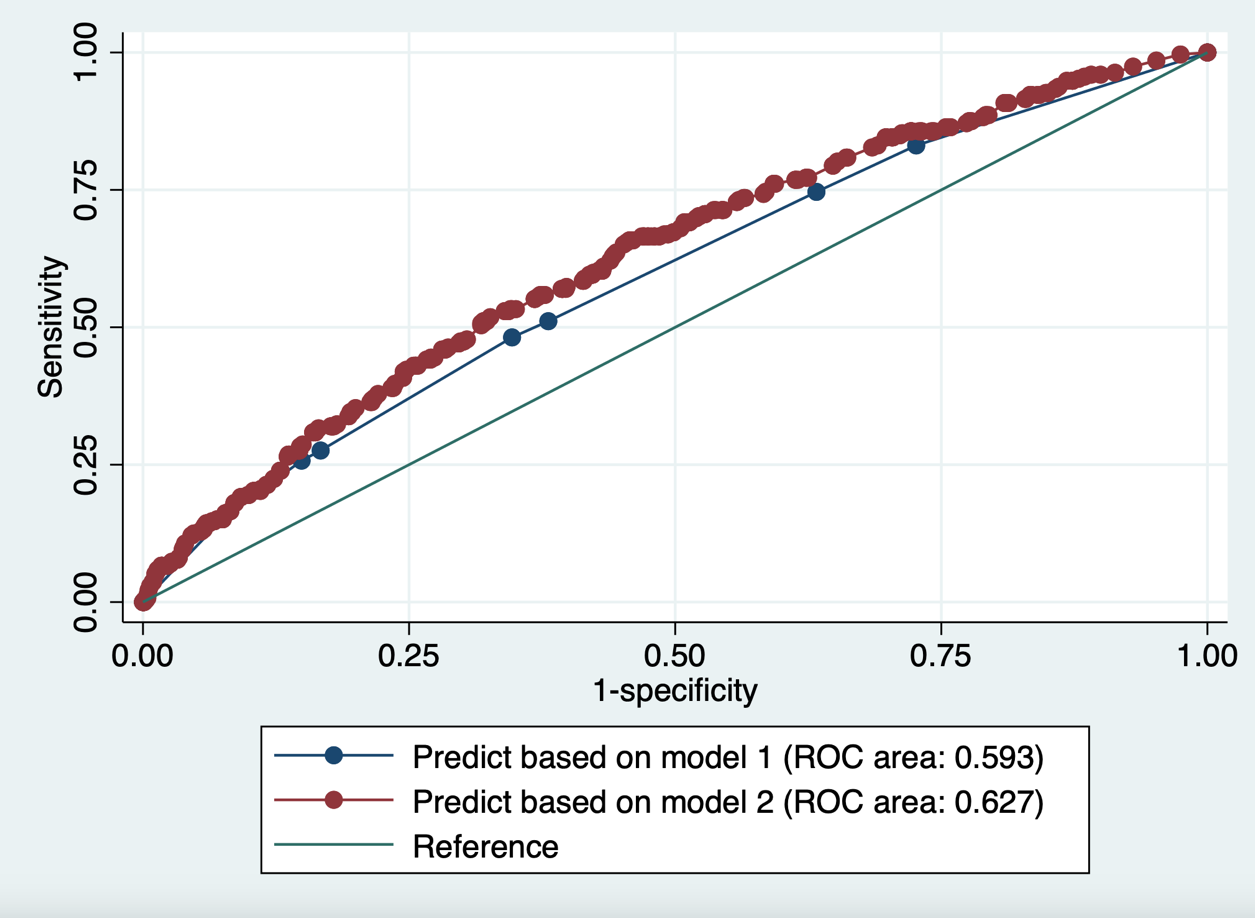


The ROC curve for Model 1 (prediction of fractures based on the responses to three questions on the physical function and falls section of the Late-Stage Elderly Questionnaire) is depicted in blue, and the ROC curve for Model 2 (prediction of fractures based on age, sex, and the responses to three questions) is depicted in red. The AUC of Models 1 and 2 were 0.594 [0.560–0.628] and 0.627 [0.593–0.661], respectively. The NRI was 0.314; 95%CI, 0.194–0.434; P< 0.001.

Abbreviations: AUC, area under the curves; NRI, net reclassification improvement; ROC, receiver operating characteristic
